# IL-33 treatment attenuated diet-induced hepatic steatosis but aggravated hepatic fibrosis

**DOI:** 10.18632/oncotarget.9259

**Published:** 2016-05-09

**Authors:** Yinjie Gao, Yuan Liu, Mei Yang, Xiaodong Guo, Min Zhang, Hanwei Li, Jin Li, Jingmin Zhao

**Affiliations:** ^1^ Department of Pathology and Hepatology, Beijing 302 Hospital, Beijing, China; ^2^ Department of Infectious Diseases, Medical School of Chinese PLA, Beijing, China; ^3^ Department of Liver Cirrhosis, Beijing 302 Hospital, Beijing, China; ^4^ Department of Liver Transplantation and Research Center, Beijing 302 Hospital, Beijing, China

**Keywords:** interleukin-33, high-fat diet, methionine-choline-deficient diet, steatosis, fibrosis, Pathology Section

## Abstract

The aim of our work was to investigate the role of interleukin-33 (IL-33) and its receptor ST2 in the progression of diet-induced nonalcoholic steatohepatitis (NASH) in mice, and the characteristic expression in livers of patients with NASH. Mice were fed with high-fat diet (HFD) or methionine-choline 4-deficient diet (MCD) and injected intraperitoneally with IL-33. Both mRNA and protein expression levels of IL-33 and ST2 were up-regulated in the livers of mice fed with HFD or MCD. Treatment with IL-33 attenuated diet-induced hepatic steatosis and reduced activities of ALT in serum, as well as ameliorated HFD-induced systemic insulin resistance and glucose intolerance, while aggravated hepatic fibrosis in diet-induced NASH. Furthermore, treatment with IL-33 can also promote Th2 response and M2 macrophage activation and beneficial modulation on expression profiles of fatty acid metabolism genes in livers. ST2 deficiency did not affect hepatic steatosis and fibrosis when fed with controlling diet. IL-33 did not affect diet-induced hepatic steatosis and fibrosis in ST2 knockout mice. Meanwhile, in the livers of patients with NASH, IL-33 was mainly located in hepatic sinusoid, endothelial cells, and hepatic stellate cells. The mRNA expression level of IL-33 and ST2 was elevated with the progression of NASH. In conclusion, treatment with IL-33 attenuated diet-induced hepatic steatosis, but aggravated hepatic fibrosis, in a ST2-dependent manner.

## INTRODUCTION

Nonalcoholic Steatohepatitis (NASH), as a progressive form of nonalcoholic fatty liver disease, leads to progressive fibrosis, cirrhosis and even hepatocellular carcinoma [1–3]. A “two-hit” model has been hypothesized as a potential mechanism responsible for NASH pathogenesis. The first “hit” is provided by metabolic syndrome (steatosis), and then the second “hit” triggers a cascade of events leading to cell death and fibrosis [4]. Inflammation is believed as the driving force of NASH and the progression to fibrosis and subsequent cirrhosis [5]. Innate immune cells, such as blood-derived monocytes and liver resident macrophages, which are termed as Kupffer cells, are likely to play a pivotal role in responding to inflammation and metabolic stresses by orchestrating local immune responses through the secretion of cytokines and chemokines [5]. T-bet, a regulator of the inflammatory T helper1 (Th1) response, was up-regulated, whereas GATA-3, a regulator of the anti-inflammatory Th2 response, was significantly down-regulated in methionine-choline-deficient-diet (MCD)-induced NASH [6, 7]. In addition, high-fat diet (HFD) has reduced hepatic NKT cells and enhanced production of Th1-type cytokines, including IL-12, IFN-γ and TNF-α [8]. Therefore, it is postulated that a Th1/Th2 balance may play an important role in the development of inflammation of NASH.

IL-33, a member of the IL-1 family, which mediates its biological effects *via* IL-1 receptor ST2, drives production of Th2-associated cytokines from *in vitro* polarized Th2 cells and induces the expression of IL-4, IL-5, and IL-13 *in vivo* [9]. IL-33 reduced HFD-induced macrophage foam cell formation and attenuated the development of atherosclerosis [10, 11]. In obese diabetic (*ob/ob*) mice, IL-33 induced accumulation of Th2 cells in adipose tissue and polarization of adipose tissue macrophages toward M2, and reduced adiposity and fasting glucose [12]. In livers, IL-33 appeared to be hepatoprotective against Con A and ischemia-reperfusion injury [13, 14], and acted as a potent immune stimulator and a hepatoprotective cytokine in acute viral hepatitis [15].

Miller et al. reported that treatment of male obese (*ob/ob*) mice (6-week-old) with recombinant IL-33 for 3 weeks reduced adiposity, but had no significant effect on liver histology or hepatic function [12]. Mice lacked of leptin (*ob/ob*) are obese, hyperphagic, and insulin resistant, and develop hepatic steatosis and type II diabetes. Although the metabolic abnormalities resemble nonalcoholic fatty liver disease, spontaneous development of steatohepatitis is not a feature of *ob/ob* mice strains [16], and in Miller's study, the *ob/ob* mice did not show the elevation of serum ALT [12]. Therefore, *ob/ob* mice might be not suitable for investigation of role of IL-33 in progression of NASH. NASH develops in HFD-fed mice, and is linked to similar pathogenic factors as in humans, with steatosis and metabolic syndrome preceding the transition to steatohepatitis [17]. Rodents fed a MCD diet develop a steatohepatitis producing hepatic lesions and changes in liver redox balance, which mimics the impairment observed in patients with NASH. The major disadvantage of the MCD model is that it is associated with significant weight loss and lower glucose levels and it does not exhibit insulin resistance [18].

In this work, we employed the mice fed with MCD or HFD as animal models of NASH to firstly investigate the role of IL-33/ST2 axis in the pathogenesis of NASH.

## RESULTS

### Both HFD and MCD induced upregulation of IL-33 and ST2 expression in livers of mice

**Figure 1:**
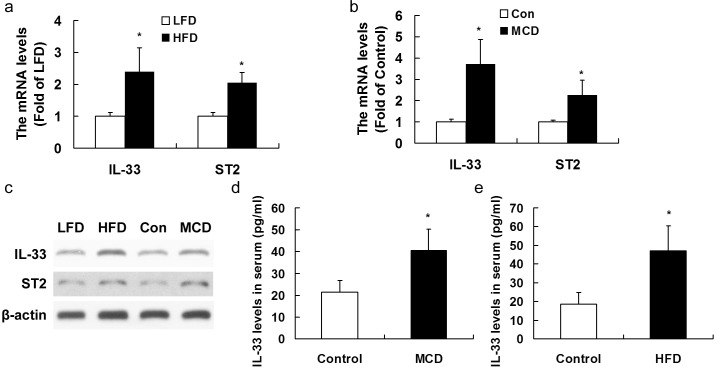
Mice were exposed to HFD for 20 weeks or MCD for 10 weeks to induce NASH Hepatic IL-33 and ST2 mRNA **a.**, **b.** and protein expression **c.** were analyzed by RT-PCR and Western blotting, respectively. IL-33 levels in serum **d.**, **e.** were analyzed by ELISA method. *n* = 8-10 in each group. Values are means ± SD; * *p* < 0.05 *versus* LFD group or control group.

We firstly analyzed the effects of a HFD diet given for 20 weeks or a MCD given for 10 weeks to mice on the expression of IL-33 and ST2. At the end of the period, mice fed on the HFD or MCD showed a significant upregulation in mRNA (Figure 1a, 1b) and protein expression levels (Figure 1c) of both IL-33 and ST2 in liver tissues. In addition, the IL-33 levels in serum (Figure 1d, 1e) were higher in mice fed with HFD or MCD than those in mice with controlling diet.

### Treatment with IL-33 attenuated HFD-induced hepatic steatosis in mice

**Figure 2:**
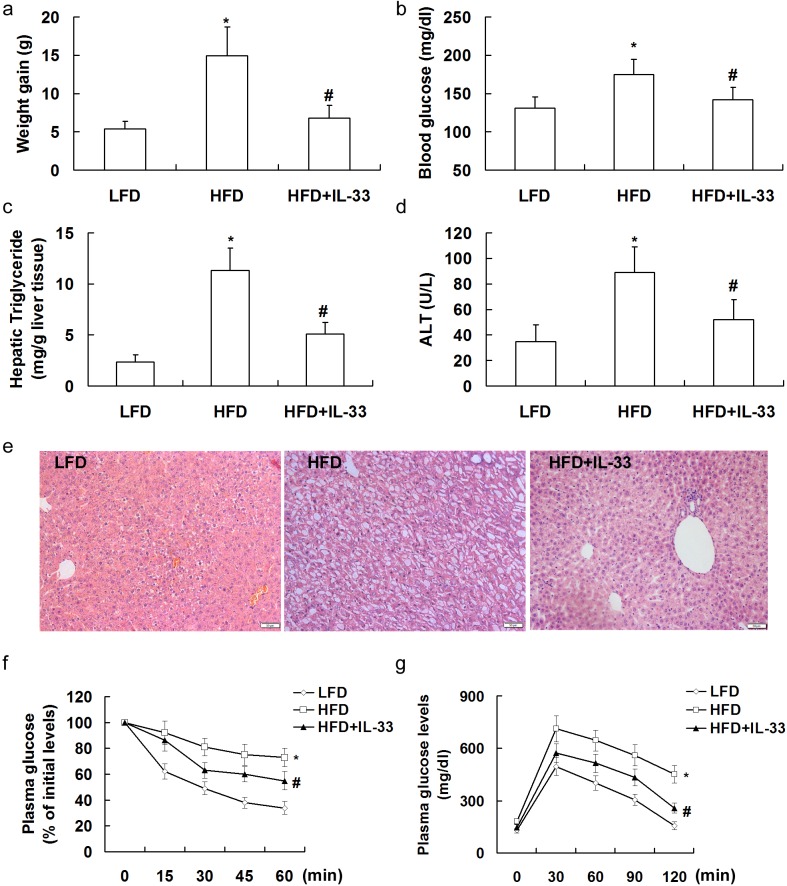
Mice were exposed to HFD for 20 weeks to induce NASH, and injected i.p twice per week with PBS or recombinant IL-33 (1 μg/injection). Graphs showed weight gain **a.**, blood glucose levels **b.**, hepatic triglyceride **c.**, and serum ALT levels **d.** in mice. Paraffin-embedded liver sections were stained with hematoxylin-eosin for evaluation of steatohepatitis **e.**. Mice were fasted for 4 h and received an intraperitoneal injection of insulin (1 U/kg body weight) **f.** or glucose (2 g/kg body weight) **g.** for insulin tolerance tests and glucose tolerance tests, respectively. *n* = 8-10 in each group. Values are means ± SD; * *p* < 0.05 *versus* LFD group; # *p* < 0.05 *versus* HFD group.

Mice were fed with HFD for 20 weeks, and injected i.p. twice per week with recombinant IL-33 (1 μg/injection). At the end of the treatment period, mice fed on the HFD showed a marked and significant increase in weight gain (Figure 2a), blood glucose levels (Figure 2b), hepatic triglyceride (Figure 2c), and serum ALT levels (Figure 2d). Treatment with IL-33 led to a significant reduction in weight gain, blood glucose levels, hepatic triglyceride, and serum ALT levels.

Hematoxylin and eosin staining (Figure 2e) revealed hepatic steatosis and ballooning degeneration of hepatocytes in the liver tissues of HFD-fed mice, which was attenuated by treatment with IL-33.

In addition, IL-33-treated mice showed a significant decrease in the severity of HFD-induced insulin resistance and glucose intolerance (Figure 2f and 2g), which were indicated by changes in plasma levels of glucose in response to a peritoneal injection of insulin and glucose, respectively. Thus, treatment with IL-33 ameliorated HFD-induced systemic insulin resistance and glucose intolerance.

### Treatment with IL-33 attenuated MCD-induced hepatic steatosis in mice

**Figure 3:**
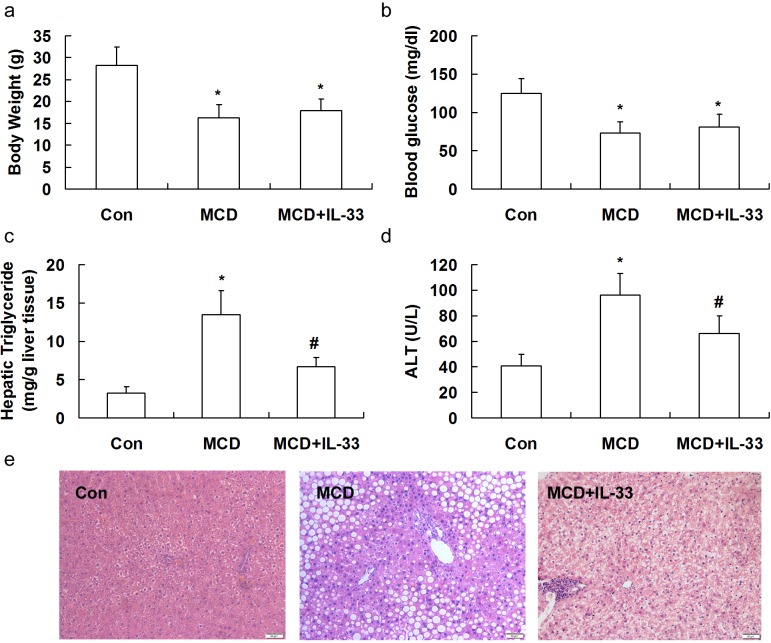
Mice were exposed to MCD for 10 weeks to induce NASH, and injected i.p twice per week with PBS or recombinant IL-33 (1 μg/injection). Graphs showed body weight **a.**, blood glucose levels **b.**, hepatic triglyceride **c.**, and serum ALT levels **d.** in mice. Paraffin-embedded liver sections were stained with hematoxylin-eosin for evaluation of steatohepatitis **e.**. *n* = 8-10 in each group. Values are means ± SD; * *p* < 0.05 *versus* control group; # *p* < 0.05 *versus* MCD group.

Mice were fed with MCD for 10 weeks, and injected i.p. twice per week with recombinant IL-33 (1 μg/injection). At the end of the treatment period, mice fed on the MCD showed a marked and significant decrease in body weight (Figure 3a) and blood glucose levels (Figure 3b), and an increased hepatic triglyceride (Figure 3c) and serum ALT levels (Figure 3d). Treatment with IL-33 had not effects on body weight and blood glucose levels, but resulted in a significant reduction in hepatic triglyceride and serum ALT levels.

Histological results indicated marked macrovesicular steatosis and ballooning degeneration in hepatocytes of MCD-fed mice, which was attenuated by treatment with IL-33 (Figure 3e).

### Treatment with IL-33 aggravated diet-induced hepatic fibrosis in mice

**Figure 4:**
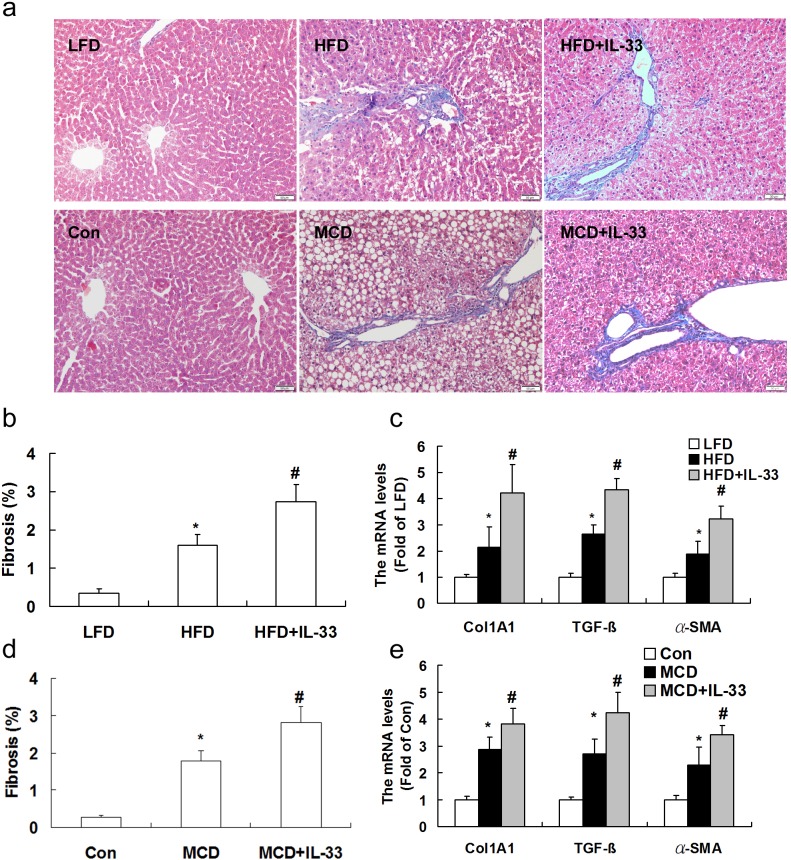
Mice were exposed to HFD or MCD, and treated with recombinant IL-33 Paraffin-embedded liver sections were Masson-trichrome-stained for evaluation of fibrosis **a.**. Graphs showed the fibrosis area **b.**, **d.** and mRNA levels of Col1A1, α-SMA and TGF-β1 **c.**, **e.** in livers. *n* = 8-10 in each group. Values are means ± SD; * *p* < 0.05 *versus* LFD group or control group; # *p* < 0.05 *versus* HFD or MCD group.

As other forms of chronic liver damage, the HFD or MCD diet triggers the initiation of a fibrogenic process that, in mice, leads to accumulation of extracellular matrix. Sections from mice fed on the HFD or MCD were stained with Masson-trichrome staining. Computerized image analysis of the slides stained with Mallory-Azan demonstrated that in HFD or MCD-fed mice showed an obvious accumulation of collagens, and treatment with IL-33 aggravated hepatic fibrosis induced by HFD (Figure 4a, 4b) or MCD (Figure 4a, 4d) in mice. Treatment of mice fed with normal diet with IL-33 (0.5 and 1 μg/injection) had no significant effect on hepatic fibrosis, hepatic triglyceride and serum ALT levels (Table S4, supplementary data). Exogenous IL-33 treatment attenuated diet-induced hepatic steatosis but exacerbated hepatic fibrosis, in a dose-dependent manner (Table S5, supplementary data). Treatment of mice fed with normal diet with exogenous IL-33 had no significant effect on ST2 protein expression in livers (Fig S1, supplementary data).

To obtain further insight into the modulation of fibrogenesis in the two models, transcript levels of genes implicated in hepatic fibrosis were evaluated. The mRNA expression of Col1A1 (Figure 4c), TGF-β1 (transforming growth factor-β1, a major pro-fibrogenic cytokine, Figure 4e), and α-SMA were significantly up-regulated by feeding the MCD or HFD. Treatment with IL-33 up-regulated expression of Col1A1, TGF-β1, and α-SMA in mice fed with HFD or MCD.

### Treatment with IL-33 promoted Th2 response and M2 macrophage activation in mice exposed to HFD or MCD

**Figure 5:**
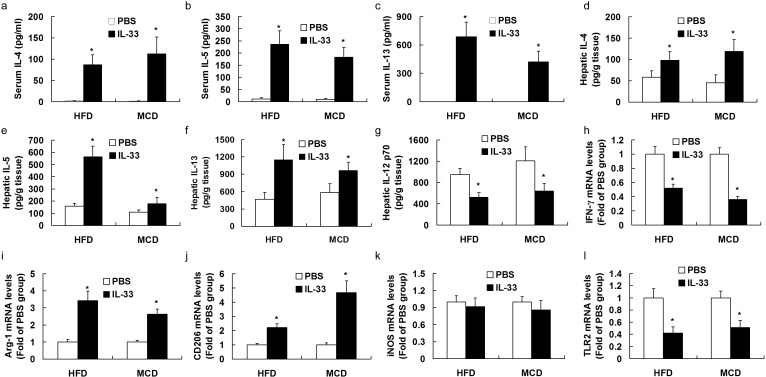
Mice were exposed to HFD or MCD, and treated with recombinant IL-33 or PBS Graphs showed the serum levels of IL-4 **a.**, IL-5 **b.**, and IL-13 **c.**, hepatic protein levels of IL-4 **d.**, IL-5 **e.**, IL-13 **f.**, and IL-12 p70 **g.**, and mRNA levels of IFN-γ **h.**, Arg-1 **i.**, CD206 **j.**, TLR2 **k.**, and iNOS **l.**. *n* = 8-10 in each group. Values are means ± SD; * *p* < 0.05 *versus* PBS-treated group.

The immunological profile of NASH mice treated with IL-33 was investigated by assessing cytokine production in serum and livers. IL-33-treated mice produced markedly more IL-4 (Figure 5a), IL-5 (Figure 5b) and IL-13 (Figure 5c) than PBS-treated mice fed with HFD or MCD, indicating that IL-33 induced accumulation of Th2 cytokines in serum. Treatment with IL-33 up-regulated proteins expression of IL-4 (Figure 5d), IL-5 (Figure 5e, and IL-13 (Figure 5f), and down-regulated IFN-γ mRNA expression (a Th1 marker, Figure 5h) in livers, indicating that IL-33 treatment induced a switch from a Th1 to a Th2 immunological profile in the livers of two experimental models of NASH.

In addition, we found that treatment with IL-33 reduced protein expression of IL-12p70 (M1 marker, Figure 5g) and mRNA expression of TLR2 (M1 marker, Figure 5l), and enhanced mRNA expression of Arg-1 and CD206 (M2 markers, Figure 5i, 5j), indicating that IL-33 treatment promoted hepatic M2 macrophage activation in experimental NASH. IL-33 treatment had no significant effect on mRNA expression of iNOS (another M1 marker, Figure 5k) in livers of two experimental models of NASH.

### Treatment with IL-33 modulated expression profiles of fatty acid metabolism genes in livers of mice exposed to MCD diet

**Figure 6:**
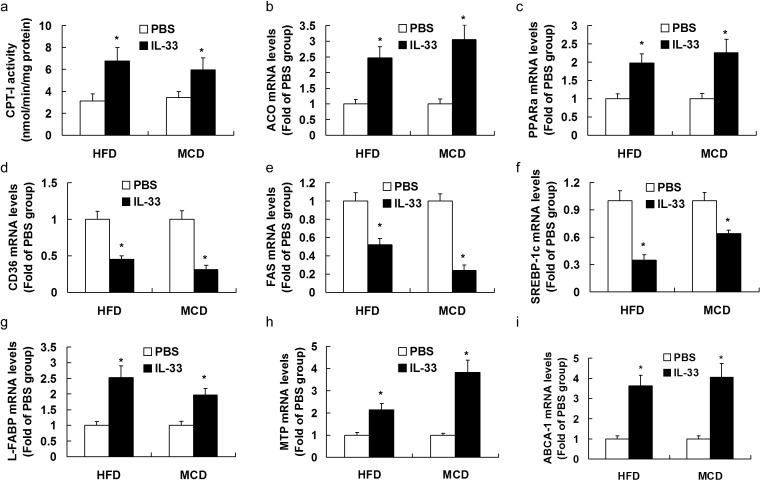
Mice were exposed to HFD or MCD, and treated with recombinant IL-33 or PBS Graphs showed the activity of CPT-I **a.**, and mRNA levels of ACO **b.**, PPARα **c.**, CD36 **d.**, FAS **e.**, SREB-1c **f.**, L-FABP **g.**, MTP **h.**, and ABCA-1 **i.** in livers. *n* = 8-10 in each group. Values are means ± SD; * *p* < 0.05 *versus* PBS-treated group.

In diet-induced NASH mice, IL-33 treatment enhanced activity of CPT-I (Figure 6a) and mRNA expression of PPARα (Figure 6b), ACO (Figure 6c), L-FABP (Figure 6g), MTP (Figure 6h) and ABCA-1 (Figure 6i), and reduced mRNA expression of CD36 (Figure 6d), FAS (Figure 6e), and SREBP-1c (Figure 6f).

### ST2 deficiency abolished the effects of IL-33 treatment

**Figure 7:**
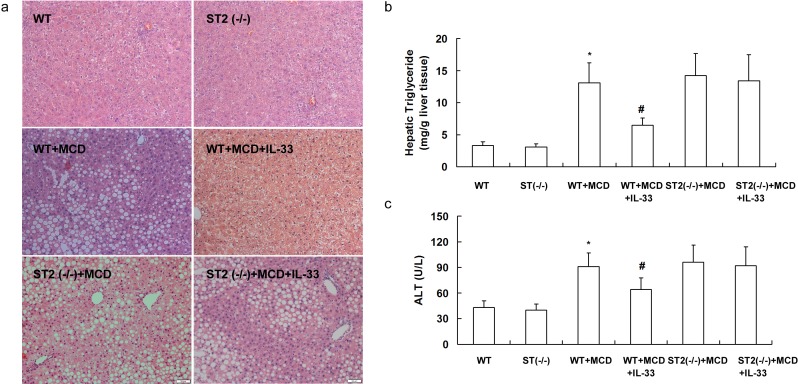
ST2 knockout mice and wild-type mice were fed with MCD, and treated with recombinant IL-33 for 10 weeks Paraffin-embedded liver sections were stained with hematoxylin-eosin for evaluation of steatosis **a.**. Graphs showed hepatic triglyceride **b.** and serum ALT levels **c.** in mice. *n* = 8-10 in each group. Values are means ± SD; * *p* < 0.05 *versus* wild-type mice fed with controlling diet; # *p* < 0.05 *versus* wild-type mice fed with MCD diet.

To confirm that IL-33 exhibited its function through its receptor ST2, ST2 knockout mice were fed with MCD for 10 weeks or HFD for 20 weeks and treated with IL-33 simultaneously. ST2 deficiency did not influence hepatic steatosis (Figure 7a), hepatic triglyceride content (Figure 7b), and serum ALT levels (Figure 7c) when fed with controlling diet.

**Figure 8:**
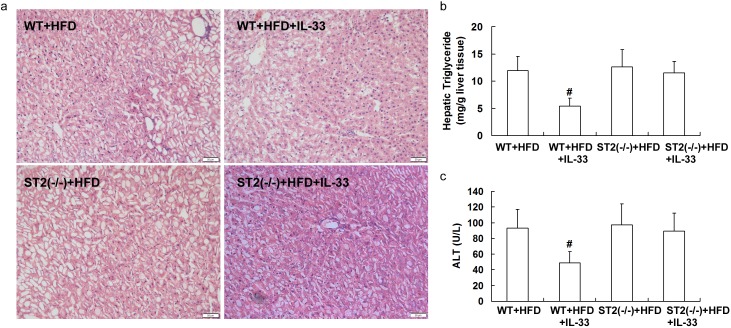
ST2 knockout mice and wild-type mice were fed with HFD, and treated with recombinant IL-33 Paraffin-embedded liver sections were stained with hematoxylin-eosin for evaluation of steatohepatitis **a.**. Graphs showed hepatic triglyceride **b.** and serum ALT levels **c.** in mice. *n* = 8-10 in each group. Values are means ± SD; # *p* < 0.05 *versus* wild-type mice fed with HFD diet.

When fed with MCD or HFD, treatment with IL-33 attenuated hepatic steatosis (Figure 7a and Figure 8a) and reduced hepatic triglyceride levels (Figure 7b and Figure 8b) and serum ALT levels (Figure 7c and Figure 8c) in wild-type mice, but had no significant effect on hepatic steatosis, hepatic triglyceride levels and serum ALT levels in ST2 knockout mice.

Computerized image analysis of the slides stained with Mallory-Azan demonstrated that, ST2 deficiency did not impact hepatic fibrosis when fed with controlling diet, as shown in supplementary data (Figure S1). When fed with HFD, treatment with IL-33 aggravated hepatic fibrosis in wild-type mice, but did not affect fibrosis in ST2 knockout mice.

### IL-33 levels in NASH patients

**Figure 9:**
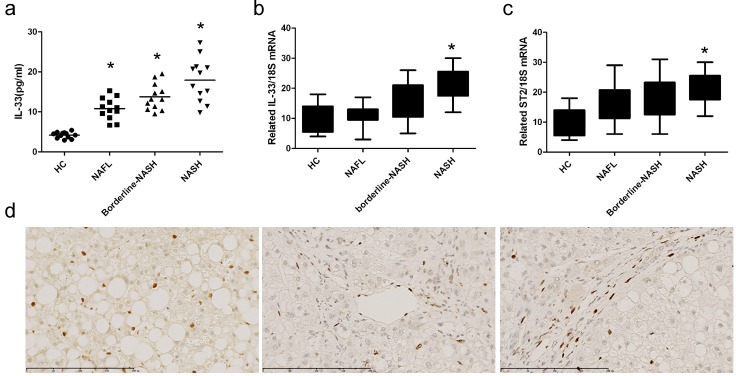
IL-33 levels in NASH patients Serum IL-33 levels **a.** were determined by ELISA method. The mRNA expression of IL-33 **b.** and ST2 **c.** was determined by RT-PCR method. Immunohistochemistry analysis of IL-33 **d.** in livers of NASH patients. HC, healthy control (*n* = 15); NAFL, non-alcoholic fatty liver (*n* = 17); borderline-NASH, borderline non-alcoholic steatohepatitis (*n* = 15); NASH, non-alcoholic steatohepatitis (*n* = 14). Values are means ± SD; * *p* < 0.05 *versus* healthy control.

General information of NASH patients were shown in supplementary data (Table S6). When compared to healthy controls, IL-33 levels (Figure 9a) were higher in serum of patients with NAFL, borderline-NASH, and NASH. Compared with healthy controls, mRNA expression of IL-33 (Figure 9b) and ST2 (Figure 9c) was higher in livers of patients with NASH. The analysis of immunohistochemical results (Figure 9d) showed that IL-33 in liver was located in hepatic sinusoid, endothelial cells, and hepatic stellate cells.

## DISCUSSION

The major finding of our study was that IL-33 treatment attenuated diet-induced hepatic steatosis and reduced serum ALT levels, but aggravated hepatic fibrosis.

Although IL-33 treatment did not affect serum cholesterol, HDL-c, VLDL/LDL-c, or triglyceride [11] in HFD-fed mice, it impaired fasting glucose and improved glucose and insulin tolerance [12]. We also found impaired fasting glucose levels and improved glucose and insulin tolerance following 20-weeks IL-33 treatment in HFD-fed mice. It was therefore that reduction in glucose levels might contribute to the protection of IL-33 against HFD-induced NASH. In this work, treatment with IL-33 also attenuated hepatic steatosis and reduced ALT levels in mice fed with MCD diet, indicating that the function of IL-33 was not totally dependent on its modulation in glucose metabolism.

IL-33 induced Th2 cytokines and Th2 antibodies in serum and lymph node cells of ApoE (−/−) mice [11], which was a classic murine model of atherosclerosis. Inflammatory process in NASH and atherosclerosis may share common mechanisms, involving the local presence of activated macrophages [19]. An increase in M1 cytokines is also associated with the development of NASH in both experimental animals and humans [20–22]. Treatment of *ob/ob* mice with IL-33 strongly enhanced the mRNA expression of M2 markers in liver, including L-arginase (Arg1) and Chi313 [12]. In this work, treatment of NASH mice with IL-33 enhanced expression of Th2 cytokines (IL-4, IL-5 and IL-13) and M2 markers (Arg-1 and CD206), and reduced Th1 cytokines (IFN-γ) and M1 markers (IL-12p70 and TLR2), indicating that IL-33 promoted Th2 cytokine synthesis leading to the polarization of liver macrophages/Kupffer cells toward an M2 phenotype, and ultimately shifted the cytokine imbalance towards anti-inflammation, which might be beneficial for reversing hepatic steatosis.

Meanwhile, it was reported that treatment with IL-33 decreased the uptake of AcLDL and OxLDL and enhanced cholesterol efflux by THP-1 macrophages through modulating expression of key genes implicated in the uptake and efflux of cholesterol including SR-A, CD36, SR-BI, ApoE, ABCA-1, and ABCG-1 [10]. Interestingly, in this work, treatment with IL-33 had a beneficial modulation on expression profiles of fatty acid metabolism genes including ACO, PPARα, CD36, FAS, SREB-1c, L-FABP, MTP, and ABCA-1 in livers. PPARα was essential in the modulation of lipid transport and metabolism, mainly through activating mitochondrial and peroxisomal fatty acid β-oxidation pathways, and liver PPARα was crucial for whole-body fatty acid homeostasis and was protective against non-alcoholic fatty liver disease [23]. L-FABP, transcriptionally regulated by PPARα, was responsible for intracellular trafficking of long chain fatty acids [24]. MTP was involved in hepatic secretion of triglyceride-rich VLDL [25]. ABCA1, mediated the transport of cholesterol and phospholipids from cells to HDL apolipoproteins, was associated with lipid storage in hepatocytes in NASH [26]. CD36, the class B scavenger receptor, has been reported to facilitate the uptake of long chain fatty acids by endothelial cells as well as macrophages, and plays a role in hepatic steatosis induced by feeding a HFD [27]. Fas, which was regulated by SREBP-1c, was an enzyme necessary for *de novo* synthesis of fatty acids [28, 29]. Therefore, the modulation of IL-33 on these genes might contribute to the beneficial effect of IL-33 against hepatic steatosis in mice fed with MCD or HFD.

However, in this work, we found that treatment with IL-33 enhanced hepatic fibrosis in mice fed with HFD. Similar result was also found by Jeftic et al [30]. Steatosis is thought to be a prerequisite for NASH and has also been identified as an independent risk factor for liver fibrosis. It was reported that inhibiting triglyceride synthesis improved hepatic steatosis but exacerbated liver damage and fibrosis in obese mice with NASH [31], so there was no cause-effect relationship between steatosis and fibrosis. IL-33 has been suggested to possess pro-fibrotic properties in skin and lung [32, 33]. IL-33 deficiency ameliorated experimental fibrosis induced by thioacetamide or carbontetrachloride in livers and hepatic IL-33/ST2 axis promoted hepatic stellate cells activation and triggered a potent fibrogenic response [34]. Some of the stereotypical Th2-associated genes including IL-4 and IL-13 may more accurately be viewed as dual role factors coordinating host defenses as well as tissue remodeling [35]. The pro-fibrotic properties of IL-33 might be detrimental and drives pathological tissue remodeling in the liver. In our work, treatment with IL-33 (1μg/injection, twice a week, 10 weeks) had no significant effect on hepatic fibrosis and serum ALT levels in mice fed with controlling diet. However, in the report of McHedlidze et al., IL-33 vector-injected mice showed excessive immune cell infiltrates and increased hepatic collagen [34]. Further investigation might be required to investigate the role of IL-33 in fibrosis in NASH.

Furthermore, it has been demonstrated that IL-33 treatment attenuated atherosclerosis in ApoE^−/−^ mice and reduced adiposity in *ob/ob* mice in a ST2 dependent manner [11, 12]. In this work, ST2 deficiency abolished the effects of IL-33 treatment on hepatic steatosis and fibrosis, so we might also conclude that the function of IL-33 in NASH mice was dependent on ST2 signaling. However, ST2 deficiency did not aggravate the steatosis or attenuated fibrosis in NASH mice. Recently, it was reported that deficiency of the endogenously produced IL-33 and its receptor ST2 did not impact the development of atherosclerosis in ApoE-deficient mice [35], which might contribute to the conclusion that the deficiency of endogenous IL-33 signaling had no impact on the Th1/Th2 cytokine profile.

In conclusion, IL-33 treatment attenuated both diets-induced hepatic steatosis, but aggravated hepatic fibrosis, in a ST2-dependent manner. The present study firstly provides evidence of dual roles for IL-33/ST2 axis in diet-induced NASH in mice.

## MATERIALS AND METHODS

### Animals

C57BL/6J mice (male, 6-week-old) were obtained from the Vital-Aiver Animal Ltd (Beijing, China). C57BL/6 ST2 knockout (ST2 ^−/−^) mice [36] were backcrossed for at least 10 generations. Mice were bred and housed in our animal facilities. All the animals were entrained to controlled temperature (23-25°C), 12-h light and 12-h dark cycles (light, 08:00-20:00 h; darkness, 20:00-08:00 h), and free access to food and tap water. All the animals used in this work received humane care in compliance with institutional animal care guidelines, and were approved by the Local Institutional Committee. All the surgical and experimental procedures were according to the criteria outlined in the “Guide for the Care and Use of Laboratory Animals” prepared by the National Academy of Sciences and published by the National Institutes of Health (NIH publication 86-23 revised 1985).

Mice were bred in-house in a pathogen-free facility and fed either a low-fat diet (LFD) or a high-fat diet (HFD, Composition of diet was shown in supplementary data, Table S1) ad libitum for 20 weeks. At the same time, the mice were injected i.p. twice per week with PBS or murine recombinant IL-33 (IL-33, 0.5 and 1 μg/injection) [11, 12].

Mice were bred in-house in a pathogen-free facility and fed either controlling diet or a Methionine and Choline Deficient Diet (MCD, Composition of diet was shown in supplementary data, Table S2) ad libitum for 10 weeks. At the same time, the mice were injected i.p. twice per week with PBS or murine recombinant IL-33 (1 μg/injection, Sigma, St Louis, MO, USA).

At the end of the experiment, all mice were sacrificed under sodium pentobarbital anesthesia and the livers were carefully removed (*n* = 17-20 total in each group).

Details of real-time quantitative polymerase chain reaction (RT-PCR), western blotting, biochemical analysis, and histological analysis were shown in supplementary data.

### Insulin and glucose tolerance tests

Mice were fasted for 4 h and received an intraperitoneal injection of insulin (1 U/kg body weight) or D-glucose (2 g/kg body weight). For insulin tolerance tests, blood samples (5 μl) were collected from the tail vein before and at 15, 30, 45, and 60 min after the bolus insulin injection. Similarly, for glucose tolerance tests, blood samples were collected from the tail vein before and at 30, 60, 90 and 120 min after the glucose bolus injection [37]. The levels of plasma glucose were measured.

### Measurement of cytokines

IL-4, IL-5, IL-12p70, IL-13, and TNF-α in livers or serum were determined by using an enzyme-linked immunosorbent assay kit (R&D Systems, MN, USA).

### Measurement of CPT-I activity

CPT-I activity was determined in livers as the incorporation of radiolabelled carnitine into acylcarnitine according to Priore et al [38].

### Patients with NASH

All patients provided the written informed consents for their blood samples, liver biopsy and clinical records to be used in this study, and the information was anonymized and de-identified prior to analysis. The study protocol was approved by the Medical Ethics Committee of the 302 Hospital, Beijing, China, and adhered to the Declaration of Helsinki.

Percutaneous liver biopsy was performed by experienced physicians with a 16-gauge Hepafix needle under the guidance of B- ultrasound. The liver biopsy specimens were immediately fixed in 10% formalin and embedded in paraffin. The histological slides were read and semiquantitatively scored by two experienced pathologists. Histological grading and staging of NASH were scored according to NASH activity score (NAS), a semi-quantitative scoring system reported by Kleiner and his colleagues [39] (details shown in supplementary data).

### Statistical analysis

The data are expressed as the mean ±standard deviation (SD). Comparison among groups was analyzed using a two-way analysis of variance followed by Bonferroni *t*-test. Significance was defined as a *P* value less than 0.05. Statistical analysis was performed using SPSS 12.0.0 software (SPSS Inc., Chicago, IL, USA).

## SUPPLEMENTARY MATERIAL TABLES AND FIGURES




