# Supplementary material for: IL-33 treatment attenuated diet-induced hepatic steatosis but aggravated hepatic fibrosis
Source: Oncotarget. 2016 May 9;7(23):33649–61. doi: 10.18632/oncotarget.9259 (PMC5085109; doi:10.18632/oncotarget.9259)
Supplement: Supplementary file 1 [file oncotarget-07-33649-s001.pdf]

IL-33 treatment attenuated diet-induced hepatic steatosis but aggravated hepatic fibrosis

Supplementary Material

Methods

Histological grading and staging of NASH patients

NAS, ranging from 0–8, was the sum of steatosis, lobular inflammation and hepatocellular ballooning scores. Steatosis was scored from 0 to 3: S0: no steatosis or less than 5%, S1: 5–33%, S2: 34–66%, S3: >66%. Lobular inflammation was graded as follows: stage 0, no foci; stage 1: < 2 foci per 200 × field; stage 2: 2–4 foci per 200 × field; stage 3: > 4 foci per 200 × field. Ballooning degeneration of liver cells was evaluated as: grade 0, absent; grade 1, few; grade 2, a lot. For analysis in this study, cases with score>5 were considered as NASH patients and cases with score<4 were grouped as non-NASH patients including simple fatty liver cases (NAFL, score: 0–2) and borderlineNASH cases (score: 3–4). The analysis was performed by investigators (Hanwei Li and Jin Li) blinded to the experimental groups.

Real-time quantitative polymerase chain reaction (RT-PCR)

Total RNA was extracted from mouse liver by homogenization in TRI reagent (Molecular Research Center, Cincinnati, OH, USA), followed by chloroform extraction and ethanol precipitation. RNA was incubated with DNase (Qiagen, Inc., Valencia, CA, USA) to remove contaminating DNA; the enzyme was then inactivated and removed according to the manufacturer’s specifications (RNeasy, Qiagen). cDNA was synthesized from 1 μg RNA in a reaction mixture containing 2.5 U/μl M-MLV reverse transcriptase (Invitrogen, Carlsbad, CA, USA) and 5 μM random hexamer primers (Invitrogen). PCR was performed with an ABI Prism 7900HT Sequence detection system (Applied Biosciences, Warrington, UK) using Power SybrGreen (Applied Biosciences, Warrington, UK). Each sample was measured intriplicate and ΔC_T values were normalized to 18S as a housekeeping gene control. The sequences of primers are listed in Table S3 (supplementary data).

Western blotting

Equal amount of protein preparations (20 μg/μl) were run on SDS-polyacrylamide gels, electrotransferred to polyvinylidine difluoride membranes, and blotted with a primary antibody against IL-33 and ST2 (Abcam, Cambridge, UK) overnight at 4°C using slow rocking. Then, they were blotted with responding HRP-conjugated secondary antibody and HRP-conjugated monoclonal antibody against β-actin. Immunoreactive bands were detected by a chemiluminescent reaction (ECL kit, Beyotime Institute of Biotechnology, Shanghai, China), and results were expressed as the ratio of the density of specific bands to the corresponding β-actin.

Biochemical analysis

Glucose levels of serum were determined spectrophotometrically using the Trinder assay (Sigma, St Louis, MO, USA). Triacylglycerols levels in livers were determined by using a MaxDiscovery™ Triglycerides Enzymatic Assay Kit (Bioo, Texas, USA). Alanine aminotransferase (ALT) in serum was determined according to the enzymatic kinetic method by using an automatic biochemical analyzer (Olympus UA2700, Tokyo, Japan).

Histological analysis

Liver tissue samples were fixed with 4% paraformaldehyde, embedded in paraffin, and stained with hematoxylin and eosin (H&E). Hepatic fibrosis was analyzed by Masson-trichrome staining. Images were captured using a BX60 camera (Olympus, Japan). The analysis was performed by investigators (Hanwei Li and Jin Li) blinded to the experimental groups.

Table S1: Composition of low-fat diet (LFD: heat of combustion 17.0KJ/g) and high-fat diet (HFD: heat of combustion 24.3KJ/g).

Macronutrients	Low-fat diet (content; g/kg)	High-fat diet (content; g/kg)
Protein	27% calories from corn (17), wheat (63), soybean (145)	15% calories from casein (200)
Carbohydrate	60% calories from corn (34), wheat (223), soybean (148)	27% calories from cornstarch (227), maltose dextrin (132), cellulose (50)
Fat	13 % calories from corn (7), wheat (16), soybean (7), soybean oil (20)	58% calories from soybean oil (70), safflower oil (270)

Table S2: Composition of the control diet and MCD diet formulas

For Table S2, please see the attached Excel file

Table S3: Sequences of oligonucleotides used as primers

For Table S3, please see the attached Excel file

Table S4: Effect of treatment with IL-33 in mice fed with normal diet.

	Control	IL-33	
		0.5 μg	1 μg
Fibrosis (%)	0.33±0.11	0.39±0.15	0.41±0.14
Hepatic triglyceride (mg/g liver tissue)	3.46±0.82	3.11±0.95	3.37±0.69
ALT (U/L)	45±8	41±12	49±9

Mice were fed with normal diet, and treated with IL-33 (twice per week) for 10 weeks. IL-33, Interleukin-33; ALT, alanine aminotransferase; n=8-10 in each group. Values are means ± SD

Table S5: Effect of treatment with IL-33 in mice fed with HFD or MCD.

	vehicle	IL-33	
		0.5 μg	1 μg
HFD			
Fibrosis (%)	1.59±0.29	2.15±0.38*	2.74±0.45*
Hepatic triglyceride (mg/g liver tissue)	11.3±2.2	6.9±1.5*	5.1±1.1*
ALT (U/L)	89±20	64±17*	52±16*
MCD			
Fibrosis (%)	1.79±0.28	2.32±0.39*	2.82±0.42*
Hepatic triglyceride (mg/g liver tissue)	13.5±3.1	7.5±2.4*	6.7±1.2*
ALT (U/L)	96±17	70±18*	66±14*

Mice were fed with HFD and treated with IL-33 (twice per week) for 20 weeks. Mice were fed with MCD and treated with IL-33 (twice per week) for 10 weeks. n=8-10 in each group. Values are means ± SD. *P<0.05 versus vehicle-treated group.

Table S6: General information of patients

	Health Control	NAFL (NAS0~2)	Borderline NASH (NAS3~4)	NASH (NAS≥5)
Number	15	17	15	14
Age	30.2±6.4	41.5±8.9	32.6±13.1	35.8±15.4
Gender (male/female)	8/7	10/7	7/8	14/10
BMI (kg/m^2)	19.1±2.4	23.9±3.2*	26.2±2.9*	29.1±3.3*
ALT (U/L)	24.3±6.5	56.4±24.1*	88.2±31.9*	165.0±30.5*
AST (U/L)	21.7±5.2	31.4±13.0	53.5±17.6*	97.6±30.4*
ALP (U/L)	87.4±15.9	93.2±18.7	109.4±16.5*	116.2±15.7*
GGT (U/L)	33.3±16.9	132.4±16.5*	89.1±12.7*	81.5±18.6*
TC (mmol/L)	4.73±0.81	4.89±0.84	4.58±0.78	4.77±1.23
TG (mmol/L)	1.83±0.49	2.31±0.67*	2.00±0.56	2.92±0.36*
HDL (mmol/L)	0.94±0.27	1.05±0.18	0.99±0.21	1.08±0.45
LDL (mmol/L)	2.84±0.37	3.57±0.69	3.32±0.61	3.22±0.54

Values are means ± SD; * $P < 0.05$ versus healthy control.

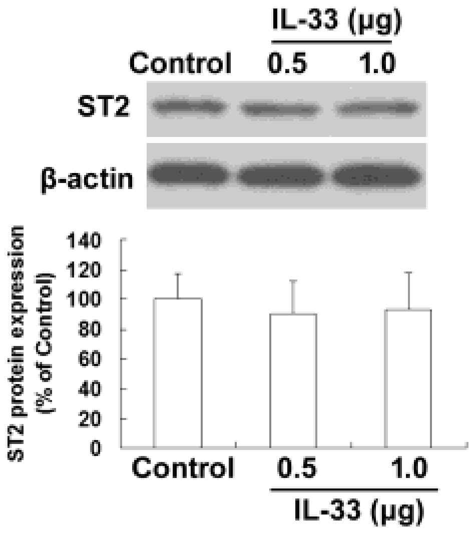

Figure S1: Effect of treatment with exogenous IL-33 on ST2 protein expression in mice fed with normal diet. Mice were fed with normal diet, and treated with IL-33 (twice per week) for 10 weeks. Values are means ± SD.

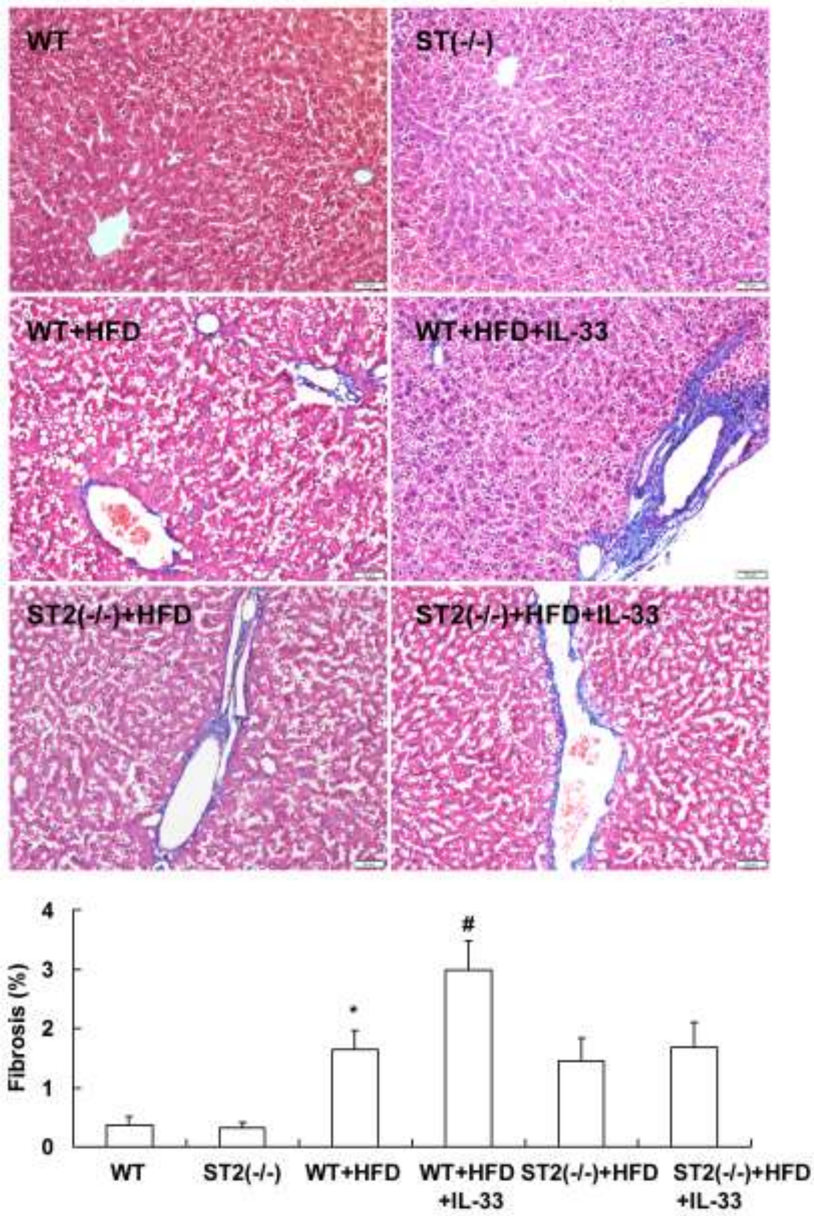

Figure S2: ST2 deficiency abolished the effect of IL-33 on hepatic fibrosis induced by HFD in mice. ST2 knockout mice and wild-type mice were fed with HFD, and treated with recombinant IL-33. Paraffin-embedded liver sections were Masson-trichrome-stained for evaluation of fibrosis. Values are means ± SD; * $P < 0.05$ versus wild-type mice fed with LFD; # $P < 0.05$ versus wild-type mice fed with HFD diet.
